# DID COVID-19 CHANGE REPORTS OF LONELINESS AND SOCIAL ISOLATION AMONG OLDER AMERICANS ACT CLIENTS?

**DOI:** 10.1093/geroni/igad104.2380

**Published:** 2023-12-21

**Authors:** Heather Menne, Claire Pendergrast

**Affiliations:** Miami University, Oxford, Ohio, United States; Syracuse University, Syracuse, New York, United States

## Abstract

COVID-19 raised immediate concern about the exacerbation of already high levels of loneliness and social isolation of older adults. Using the cross-sectional 2019 and 2021 National Survey of Older Americans Act Participants, we explored changes among selected populations of OAA clients – family caregivers (CGs), home-delivered meal clients (HDMs), and congregate meal clients (CMs). Descriptive statistics, t-tests, and review of 95% confidence intervals between years were conducted to understand rates of loneliness (UCLA 3-items) and social isolation (1-item). Results were not statistically significant but yield important findings. First, there were more cases of loneliness (48-70%) and social isolation (31-50%) than was known for the general older adult population before COVID-19. Second, the changes between 2019 and 2021 varied by service and gender. Among CGs, there was little difference in the cases of social isolation and loneliness from 2019 to 2021; however, male CGs reported a 20.3% increase in cases of social isolation. For HDMs there was a little difference over time in cases of social isolation and loneliness. There was a 23.3% increase in the number of CMs reporting social isolation and a small increase (9.3%) in CMs reporting loneliness; however, there was an 18.4% increase in cases of loneliness among male CMs. These results warrant further investigation to understand whether OAA services are a protective factor for social connections and further implementation of innovative approaches to lower overall rates of social isolation and loneliness among OAA clients.

